# B cell analysis of ethnic groups in Mali with differential susceptibility to malaria

**DOI:** 10.1186/1475-2875-11-162

**Published:** 2012-05-11

**Authors:** Silvia Portugal, Didier Doumtabe, Boubacar Traore, Louis H Miller, Marita Troye-Blomberg, Ogobara K Doumbo, Amagana Dolo, Susan K Pierce, Peter D Crompton

**Affiliations:** 1 Laboratory of Immunogenetics, National Institute of Allergy and Infectious Diseases, National Institutes of Health, Rockville, MD 20852, USA; 2 Malaria Research and Training Centre, Department of Epidemiology of Parasitic Diseases, Faculty of Medicine, Pharmacy, and Odonto-Stomatology, University of Bamako, Bamako, Mali; 3 Laboratory of Malaria and Vector Research, National Institute of Allergy and Infectious Diseases, National Institutes of Health, Rockville, MD 20852, USA; 4 Department of Immunology, Wenner-Gren Institute, Stockholm University, Stockholm, Sweden

## Abstract

**Background:**

Several studies indicate that people of the Fulani ethnic group are less susceptible to malaria compared to those of other ethnic groups living sympatrically in Africa, including the Dogon ethnic group. Although the mechanisms of this protection remain unclear, the Fulani are known to have higher levels of *Plasmodium falciparum*-specific antibodies of all Ig classes as compared to the Dogon. However, the proportions of B cell subsets in the Fulani and Dogon that may account for differences in the levels of Ig have not been characterized.

**Methods:**

In this cross-sectional study, venous blood was collected from asymptomatic Fulani (n = 25) and Dogon (n = 25) adults in Mali during the malaria season, and from *P. falciparum*-naïve adults in the U.S. (n = 8). At the time of the blood collection, *P. falciparum* infection was detected by blood-smear in 16% of the Fulani and 36% of the Dogon volunteers. Thawed lymphocytes were analysed by flow cytometry to quantify B cell subsets, including immature and naïve B cells; plasma cells; and classical, activated, and atypical memory B cells (MBCs).

**Results:**

The overall distribution of B cell subsets was similar between Fulani and Dogon adults, although the percentage of activated MBCs was higher in the Fulani group (Fulani: 11.07% [95% CI: 9.317 – 12.82]; Dogon: 8.31% [95% CI: 6.378 – 10.23]; P = 0.016). The percentage of atypical MBCs was similar between Fulani and Dogon adults (Fulani: 28.3% [95% CI: 22.73 – 34.88]; Dogon: 29.3% [95% CI: 25.06 – 33.55], but higher than U.S. adults (U.S.: 3.0% [95% CI: -0.21 - 6.164]; P < 0.001). *Plasmodium falciparum* infection was associated with a higher percentage of plasma cells among Fulani (Fulani infected: 3.3% [95% CI: 1.788 – 4.744]; Fulani uninfected: 1.71% [95% CI: 1.33 – 2.08]; P = 0.011), but not Dogon adults.

**Conclusion:**

These data show that the malaria-resistant Fulani have a higher percentage of activated MBCs compared to the Dogon, and that *P. falciparum* infection is associated with a higher percentage of plasma cells in the Fulani compared to the Dogon, findings that may account for the higher levels of *P. falciparum* antibodies in the Fulani.

## Background

Several studies have demonstrated that individuals of the Fulani ethnic group in West Africa are at lower risk of malaria and tend to have lower *Plasmodium falciparum* parasite densities compared to individuals of other ethnic groups living sympatrically with the Fulani, including the Dogon [1]. Although protective mechanisms among the Fulani remain unclear, many investigators have consistently shown that the Fulani have higher levels of antibodies specific for *P. falciparum* antigens expressed at the liver and blood stages [1-5], and enhanced IgG1 and IgG3 subclass and IgM antibody responses to malaria [6]. The B cell biology underlying these observations is not understood.

It is now well established that long-term antibody responses require the generation and maintenance of memory-B cells (MBCs) and long-lived plasma cells (LLPCs), defined in humans by the cell surface markers CD19^+^CD27^+^CD38^−^ and CD19^+^CD27^++^CD38^+++^, respectively (reviewed in [7-9]). The process of generating MBCs and LLPCs begins when naïve B cells encounter their cognate antigen near the interface of B and T cell areas of secondary lymphoid tissue, which drives naïve B cells to differentiate into isotype-switched short-lived, plasma cells (SLPCs) within the extra-follicular region, which contributes to the initial control of infections. Alternatively, naïve B cells enter follicles where germinal centers are formed, and after a period of 7–10 days, during which the CD4^+^ T-cell-dependent process of affinity maturation and immunoglobulin class-switching occurs, the germinal center reaction yields LLPCs and MBCs of higher affinity than the initial wave of SLPCs. LLPCs migrate to the bone marrow where they constitutively secrete antibody and provide a critical first line of defense against re-infection, whereas MBCs recirculate and mediate recall antibody responses after re-exposure to their cognate antigen by rapidly proliferating and differentiating into plasma cells.

Recently, it was reported that *P. falciparum* exposure in Malian children and adults, as well as Peruvian adults [10], is associated with an expansion of a phenotypically distinct population of MBCs identified as CD10^−^ CD19^+^ CD20^+^ CD21^−^ CD27^−^, similar to a MBC subpopulation initially identified in healthy US individuals in mucosal-associated lymphoid tissues by expression of the inhibitory receptor Fc-receptor-like-4 (FCRL4) [11]. B cells with a similar phenotype have been identified in individuals infected with HIV [12] and HCV [13]. Moir *et al* showed that compared to naïve B cells and classical MBCs, FCRL4^+^ MBCs proliferated less well in response to BCR-cross-linking and/or to CD40L and Toll-like receptor 9 (TLR9) agonist CpG, and showed a decreased ability to differentiate into antibody secreting cells in response to polyclonal stimulation [12]. FCRL4^+^ MBCs in HIV-viremic [12] and *P. falciparum*-exposed individuals [11] also express high levels of inhibitory receptors and a profile of lymphoid-homing receptors similar to that expressed on exhausted CD8^+^ T cells during chronic viral infections [14]. Given that the function of FCRL4^+^ MBCs in *P. falciparum*-exposed individuals is unknown, we refer to this B cell subset in the context of malaria as ‘atypical’ rather than ‘exhausted’.

Here, the proportion of peripheral blood B cells that are immature and naïve B cells; plasma cells; and classical, activated, and atypical MBCs are compared in Fulani and Dogon adults [1], and further comparisons of these B cell subsets in asymptomatic *P. falciparum* infected and uninfected individuals from both ethnic groups are presented.

## Methods

### Mali study site and participants

This cross-sectional study was done in October 2008 in Mantéourou, Mali, a rural village approximately 850 km north of the capital of Bamako. A detailed description of the study site has been published elsewhere [1]. Participants were randomly selected from an ongoing cohort study which has been described in detail elsewhere [1]. *Plasmodium falciparum* transmission is seasonal and intense at this site from July through December. The entomological inoculation was approximately 17 infective bites/person/month in September of 2000. This cross-sectional study includes 50 adults enrolled in October 2008. As is typical in Mali, the *P. falciparum*-infected adults had no signs or symptoms of malaria when the blood smear was prepared and they were not treated with anti-malarial drugs (the blood smear was not read immediately). The Ethics Committee of the Faculty of Medicine, Pharmacy, and Odonto-Stomatology at the University of Bamako, Mali approved this study. De-identified specimens were obtained from this study for immunological analyses. Written, informed consent was obtained from all study participants.

### U.S. blood donors

Peripheral blood mononuclear cells (PBMCs) from eight healthy adult blood bank donors in the U.S. were also analysed. Demographic and travel history data were not available from these anonymous donors but prior *P. falciparum* exposure is unlikely. Blood samples were obtained for research use after written informed consent was obtained from all study participants enrolled in a protocol approved by the Institutional Review Board of the National Institute of Allergy and Infectious Diseases, NIH (protocol # 99-CC-0168).

### PBMC isolation, cryopreservation, and recovery

Malian blood samples were drawn by venipuncture into sodium citrate-containing cell preparation tubes (BD, Vacutainer CPT Tubes). PBMCs were isolated according to the manufacturer's instructions and frozen in foetal bovine serum (FBS) (Gibco, Grand Island, NY) containing 7.5% dimethyl sulfoxide (DMSO; Sigma-Aldrich, St. Louis, MO). U.S. blood samples were drawn into heparinized tubes (BD) and PBMC were isolated from whole blood by Ficoll-Hypaque density gradient centrifugation (Amersham Biosciences) according to the manufacturer's instructions, and frozen in same conditions as Malian samples. PBMCs were kept at −80°C for 24 hours using freezing containers (VWR International) to control the rate of freezing and then transferred to liquid nitrogen. For analysis, PBMCs were rapidly thawed in a 37°C water bath, washed first in PBS with 10% heat-inactivated FBS and then in complete RPMI (RPMI 1640 with L-glutamine supplemented with 10% heat-inactivated FBS, penicillin/streptomycin 10,000 μg/ml, and 50 μM β-mercaptoethanol (all from GIBCO, Invitrogen)).

### B cell phenotypic analysis

PBMCs were washed in PBS with 4% heat-inactivated FBS and incubated for 30 min at 4°C with mouse monoclonal fluorescently-labeled antibodies specific for CD10 APC (HI10a) and CD20 APC-H7 (L27) (BD Biosciences); CD19 PerCP‒Cy5.5 (SJ25C1) and CD27 PE-Cy7 (O323) (eBioscience) and CD21 FITC (BL13) (Beckman Coulter). Flow cytometry was performed with a BD™ LSR II Table flow cytometer (BD Biosciences) and data were analysed using FlowJo software (Tree Star, Inc).

### Measurement of peripheral blood *P. falciparum* parasitaemia

Thick blood smears were stained with Giemsa and counted against 300 leukocytes. *Plasmodium falciparum* densities were recorded as the number of asexual parasites/μl of whole blood based on an average leukocyte count of 7,500/μl. Each smear was evaluated separately by two expert microscopists. Any discrepancies were resolved by a third expert microscopist.

### Statistical analysis

Data were analysed using GraphPad Prism 5 for Mac OS X (GraphPad Software, version 5.0d). The non-parametric Mann–Whitney test was used to compare continuous variables between groups, and the Fisher's exact test was used to compare categorical variables. For all tests, two-tailed p values were considered significant if ≤0.05.

## Results and discussion

### Study subject characteristics

Twenty-five Fulani adults and 25 Dogon adults living in the same village in Mali were included in this study. Demographic and *P. falciparum* infection status data are shown in Table 1 according to ethnic group. The average age of the Fulani participants was 38.9 years (range: 19–57 years) and 60% were female. The average age of the Dogon participants was 38.0 years (range: 21–57 years) and 36% were female. At the time of the venous blood collection, four Fulani (16%) and nine Dogon (36%) were infected with *P. falciparum* by blood-smear. Of those who were infected, the mean parasite densities for the Fulani and Dogon were 2281.25 ± 1285.9 and 2563.5 ± 1652.9 asexual parasites/μl, respectively. All participants were afebrile and asymptomatic at the time of venous blood draw.

**Table 1:** Age and gender distribution of participants by ethnic group, parasite prevalence and parasite density at the time of venous blood collection

	**Fulani**	**Dogon**	**P value**
Number of subjects	25	25	
Age-range (years)	21 - 57	22 - 57	
Age-median (years)	37	38	ns 0.777
Gender distribution (% women)	36	52	ns 0.138
Parasite prevalence (%)	16	36	ns 0.196
Parasite density +/− SD (parasites/μl)	2281.25 ± 1285.9	2563.5 ± 1652.9	ns 0.766

### Phenotypic analysis of B cell subsets

The proportion of various B cell subsets in the peripheral blood of Fulani (n = 25) and Dogon (n = 25) adults was compared by flow cytometry analysis of thawed PBMCs, which had been collected at the peak of the malaria season. These data were compared to B cell profiles of *P. falciparum*-naïve U.S. adults (n = 8).

**Figure 1:**
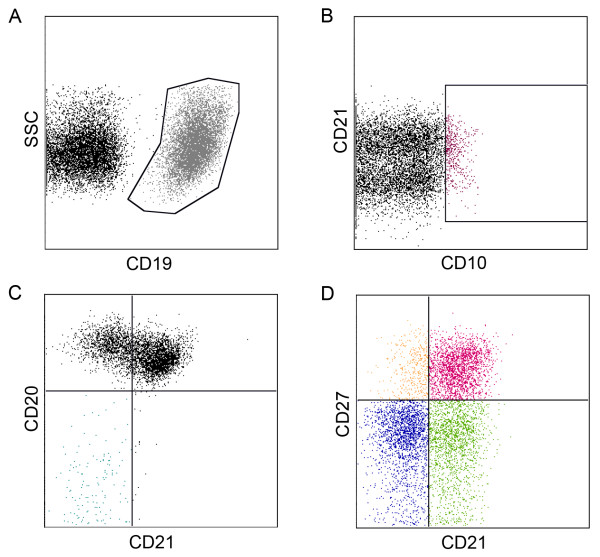
**Flow cytometry gating strategies for B cell phenotyping.** FACS plots of B cell subsets of a representative Fulani adult. Within the CD19^+^ gate shown in gray **(A)**, the B cell subpopulations are defined as follows: immature (CD10^+^) shown in purple **(B)**; plasma cells/plasma blasts (CD20^-^ CD21^-^ CD27^+^); shown in blue **(C)**; activated MBCs (CD10^-^ CD20^+^ CD21^-^ CD27^+^), classical MBCs (CD10^-^ CD20^+^ CD21^+^ CD27^+^), atypical MBCs (CD10^-^ CD20^+^ CD21^-^ CD27^-^) and naïve B cells (CD10^-^ CD20^+^ CD21^+^ CD27^-^) shown in orange, pink, blue and green respectively **(D)**.

A panel of fluorophore-conjugated antibodies specific for CD10, CD19, CD20, CD21 and CD27 allowed for the quantification of immature B cells (CD10^+^CD19^+^), naïve B cells (CD10^-^CD19^+^CD20^+^CD21^+^CD27^-^), plasma cells/plasma blasts (CD10^-^CD19^+^CD20^-^CD21^-^CD27^+^), classical MBCs (CD10^-^CD19^+^CD20^-^CD21^+^CD27^+^), activated MBCs (CD10^-^CD19^+^CD20^+^CD21^-^CD27^+^) and atypical MBCs (CD10^-^CD19^+^CD20^+^CD21^-^CD27^-^). Figure 1 shows the gating strategy used to identify these B cell subpopulations in a representative Fulani adult.

**Figure 2:**
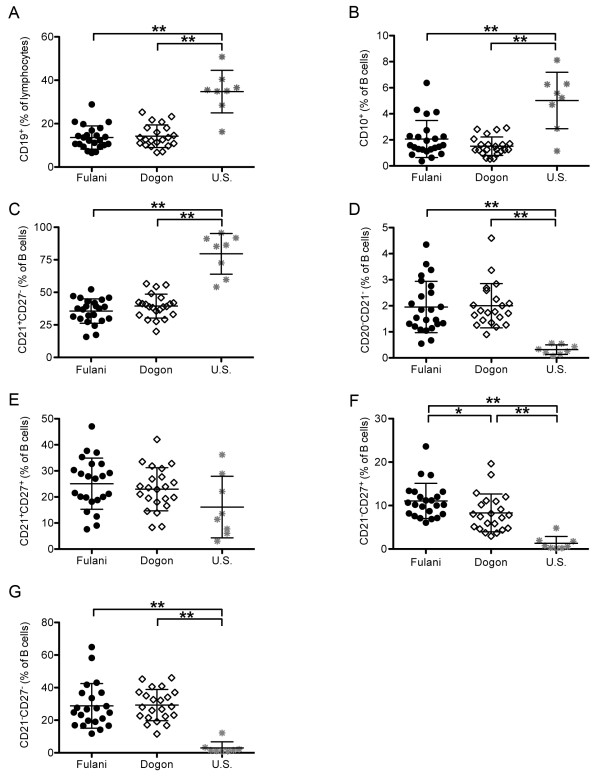
**B cell populations of Fulani, Dogon and U.S. volunteers.** PBMCs from Fulani, Dogon and U.S. volunteers were analysed by flow-cytometry. Quantitation of total B cells (CD19^+^) **(A)**, immature B cells (CD10^+^ CD19^+^) **(B)**, naïve B cells (CD21^+^ CD27^-^) **(C)**, plasma cells/plasma blasts (CD20^-^ CD21^-^) **(D)**, classical MBCs (CD21^+^ CD27^+^) **(E)**, activated MBCs (CD21^-^ CD27^+^) **(F)** and atypical MBCs (CD21^-^ CD27^-^) **(G)**. N = 25 for Fulani and Dogon volunteers and n = 8 for U.S. volunteers. Lines represent mean % of live lymphocytes; error bars indicate SD (* p < 0.05; ** p < 0.001).

As a percentage of total lymphocytes, the mean percentage of total B cells (CD19^+^) was similar in Fulani and Dogon adults (Figure 2A; Fulani: 13.6% [95% CI: 11.3 – 15.94]; Dogon: 14.2% [95% CI: 11.86 – 16.60], but significantly lower than U.S. adults (Figure 2A; U.S.: 34.8% [95% CI: 26.56 – 42.96]; p < 0.001 versus Fulani and Dogon). As a percentage of total B cells, the mean percentages of immature B cells (Figure 2B; Fulani: 2.066% [95% CI: 1.452 – 2.679]; Dogon: 1.514% [95% CI: 1.2 – 1.829] and naïve B cells (Figure 2C; Fulani: 35.6% [95% CI: 31.55 – 39.63]; Dogon: 39.4% [95% CI: 35.33 – 43.52] were also similar between the Fulani and Dogon, but lower than U.S. adults (Figure 2B and 2C; U.S. immature B cells: 5.023% [95% CI: 3.212 – 6.833]; U.S. naïve B cells: 79.6% [95% CI: 66.53 – 92.65]; p < 0.001 for all comparisons). The mean percentage of plasma cells/plasma blasts was also similar among Fulani and Dogon adults (Figure 2D; Fulani: 1.95% [95% CI: 1.537 – 2.371]; Dogon: 2.0% [95% CI: 1.616 – 2.391], but higher than U.S. adults (Figure 2D; U.S.: 0.3188% [95% CI: 0.1656– 0.4720]; p < 0.001 versus Fulani and Dogon). The mean percentage of classical MBCs did not differ significantly across the three groups (Figure 2E; Fulani: 25.08% [95% CI: 20.84 – 29.32]; Dogon: 22.97% [95% CI: 19.3 – 26.64]; U.S.: 16.12% [95% CI: 6.25 – 25.99]). Interestingly, the mean percentage of activated MBCs was significantly higher in Fulani versus Dogon adults (Figure 2F; Fulani: 11.07% [95% CI: 9.317 – 12.82]; Dogon: 8.31% [95% CI: 6.378 – 10.23]; p = 0.016) and both groups were higher than U.S. adults (Figure 2F; U.S.: 1.32% [95% CI: 0.011 – 2.63]; p < 0.001 versus Fulani and Dogon). Finally, the mean percentage of atypical MBCs was similar among Fulani and Dogon adults (Figure 2G; Fulani: 28.3% [95% CI: 22.73 – 34.88]; Dogon: 29.3% [95% CI: 25.06 – 33.55], but higher than U.S. adults (Figure 2G; U.S.: 3.0% [95% CI: -0.21 - 6.164]; p < 0.001 versus Fulani and Dogon).

Together these data indicate that the overall distribution of B cell subsets is similar among the Fulani and Dogon adults in this study population, with the exception of activated MBCs, which were increased among the Fulani. Activated MBCs are known to promote signaling cascades that induce their differentiation into antibody secreting cells, thus a higher level of activated MBCs among the Fulani is consistent with their propensity toward higher antibody responses and decreased malaria susceptibility [2,6].

The higher percentage of atypical and activated MBCs in Fulani and Dogon Malians versus U.S. adults is consistent with previous studies of *P. falciparum*-exposed individuals at separate study sites in Mali [11], in The Gambia [15], and Peru [10]. These data provide further evidence that an increase in atypical MBCs is a generalizable finding among *P. falciparum*-exposed populations. The specificity and function of atypical MBCs in the context of *P. falciparum* transmission remains to be determined. It is possible that atypical MBCs contribute to protection against malaria by regulating the host immune response; on the other hand, it is also conceivable that functional exhaustion of *P. falciparum*-specific MBCs through repeated infections contributes to the inefficient acquisition and relatively rapid loss of *P. falciparum*-specific MBCs and long-lived antibodies which has been observed in areas of intense *P. falciparum* transmission [16,17].

Also consistent with previous observations [10,11,16], it was found that relative to U.S. volunteers, Malian adults had a lower proportion of immature and naïve B cells and a higher proportion of activated MBCs and plasma cells/plasmablasts, possibly reflecting greater cumulative immunological experience.

**Figure 3:**
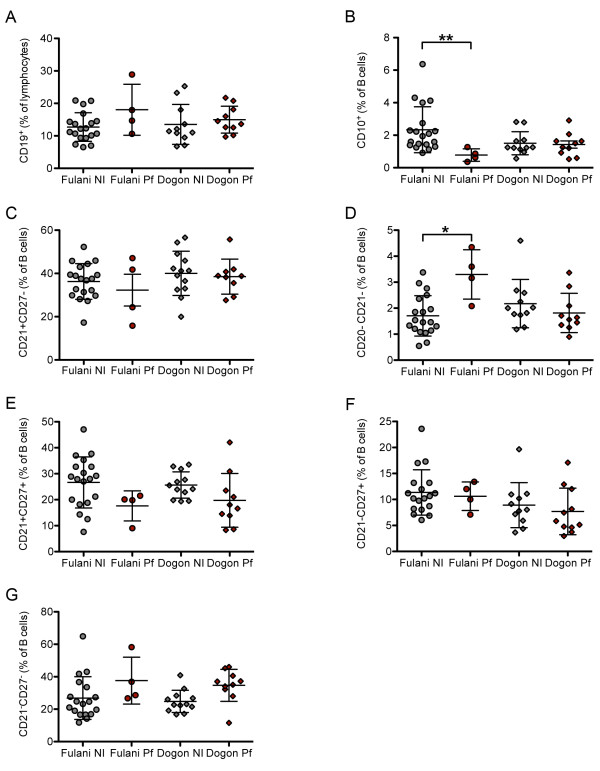
**B cell populations from *P. falciparum *-infected Fulani and Dogon volunteers. ** PBMCs from *P. falciparum*-infected Fulani, Dogon and U.S. volunteers were analyzed by flow-cytometry. Quantitation of total B cells (CD19^+^) **(A)**, immature B cells (CD10^+^ CD19^+^) **(B)**, naïve B cells (CD21^+^ CD27^-^) **(C)**, plasma cells/plasma blasts (CD20^-^ CD21^-^) **(D)**, classical MBCs (CD21^+^ CD27^+^) **(E)**, activated MBCs (CD21^-^ CD27^+^) **(F)**, and atypical MBCs (CD21^-^ CD27^-^) **(G)**. N = 4 infected Fulani; n = 9 infected Dogon. Lines represent mean % of live lymphocytes; error bars indicate SD (* p < 0.05; ** p < 0.01).

To assess whether concurrent asymptomatic *P. falciparum* infection was associated with alterations in B cell subsets, Fulani and Dogon adults were stratified by whether or not the thick blood smears were positive for *P. falciparum* infection at the time of blood collection. *Plasmodium falciparum* infection status was not associated with significant differences in the percentages of total B cells (Figure 3A). However, *P. falciparum* infection was associated with a lower percentage of immature B cells among the Fulani (Figure 3B; Fulani uninfected: 2.336% [95% CI: 1.656 – 3.016]; Fulani infected: 0.7803% [95% CI: 0.167 – 1.393]; p = 0.005), but not the Dogon (Figure 3B; Dogon uninfected: 1.505% [95% CI: 1.029 – 1.982]; Dogon infected: 1.428% [95% CI: 0.9207 – 1.935]). *Plasmodium falciparum* infection was not associated with significant differences in the percentage of naïve B cells in either ethnic group (Figures 3C), while *P. falciparum* infection was associated with a significantly higher percentage of plasma cells/plasma blasts in the Fulani (Figure 3D; Fulani uninfected: 1.71% [95% CI: 1.33 – 2.08]; Fulani infected: 3.3% [95% CI: 1.788 – 4.744]; p = 0.011), but not the Dogon (Figure 3D; Dogon uninfected: 2.17% [95% CI: 1.549 – 2.799]; Dogon infected: 1.81% [95% CI: 1.276 – 2.536]). The higher percentage of antibody-secreting plasma cells observed in *P. falciparum*-infected Fulani adults is consistent with the higher levels of *P. falciparum-*specific antibodies observed by others in this ethnic group [2-4]. Asymptomatic *P. falciparum* infection was not associated with significant changes in the percentages of classical, activated or atypical MBCs in either ethnic group (Figures 3E–G).

## Conclusion

It is well established that the Fulani ethnic group is less susceptible to malaria compared to other sympatric ethnic groups in West Africa, but the mechanisms underlying this observation remain obscure. The enhanced *P. falciparum*-specific antibody response of the Fulani group is among the more consistent observations, which led us to investigate potential differences in B cell subpopulations. Indeed, the Fulani had a higher percentage of activated MBCs compared to the Dogon, and that *P. falciparum* infection was associated with higher percentage of antibody-secreting cells among the Fulani but not the Dogon. These results serve as a basis for further studies of the B cell biology that underlies the association between Fulani ethnicity and decreased malaria susceptibility.

## Competing interests

The authors declare that they have no competing interests.

## Authors' contributions

SP designed and carried out the flow cytometry, analysed the data, and drafted the manuscript. DD participated in the design and coordination of the study in Mali and analysed the clinical data. BT, LHM, MTB, OKD and AD conceived of the study and participated in its design and coordination in Mali. SKP and PDC conceived of the study, participated in its design and coordination in Mali, and helped to analyse the data and draft the manuscript. All authors read and approved the final manuscript.
